# A heterotrimeric SMARCB1–SMARCC2 subcomplex is required for the assembly and tumor suppression function of the BAF chromatin-remodeling complex

**DOI:** 10.1038/s41421-020-00196-4

**Published:** 2020-09-22

**Authors:** Guidong Chen, Hao Zhou, Beibei Liu, Yan Wang, Jianchun Zhao, Filippo G. Giancotti, Jiafu Long

**Affiliations:** 1 State Key Laboratory of Medicinal Chemical Biology and College of Life Sciences, Nankai University, 94 Weijin Road, Tianjin 300071, China; 2 Department of Cancer Biology and David H Koch Center for Applied Research of Genitourinary Cancers, The University of Texas MD Anderson Cancer Center, Houston, TX 77230 USA

**Keywords:** X-ray crystallography, Chromatin remodelling

Dear Editor,

The SWI/SNF complex utilizes its ATP-dependent chromatin-remodeling activity to mobilize nucleosomes and thus regulates DNA accessibility at nucleosomal templates. The BAF (mammalian SWI/SNF) complex is composed of approximately 15 protein subunits^1,2^. An important subcomplex of BAF consists of one of two mutually exclusive catalytic ATPase subunits (SMARCA2/Brm and SMARCA4/Brg1), SMARCB1/BAF47/INI1/SNF5, SMARCC1/BAF155, and SMARCC2/BAF170^3^. Among them, SMARCC1 and SMARCC2 are highly similar to each other (Supplementary Fig. S1a) and considered as the key scaffold proteins in assembling and regulating the BAF^4^, which are mutated in several cancers^2,5^. Intriguingly, *SMARCB1* was found to be biallelically inactivated in ~98% of all malignant rhabdoid tumors, aggressive pediatric tumors^6,7^.

Recently, the cryo-electron microscopy (cryo-EM) structures of nucleosome bound-SWI/SNF, -RSC, or -BAF complexes were determined^8–10^. However, the assembly and structure of subcomplexes Snf5-Swi3, SMARCB1-SMARCC2, and SFH1-RSC8 present in these articles are limited. Meantime, the mechanisms through which mutations in specific BAF subunits underlie the development of different cancer types are not well known.

Firstly, we used a truncation-based approach to gradually map the minimal binding region of SMARCB1 and SMARCC2 (Supplementary Fig. S1b-g). We found that a region of SMARCB1 (aa 169–385, SMARCB1^(169–385)^), and the SWIRM domain of SMARCC2 (aa 423–518, SMARCC2^(423–518)^) (Fig. 1a) form a stable subcomplex (Supplementary Fig. S1g). We successfully crystallized a complex comprising a SMARCC2 fragment SMARCC2^(325–518)^ and SMARCB1^(169–385)^ (Fig. 1a and Supplementary Fig. S1h). Finally, we determined the crystal structure of the human SMARCB1–SMARCC2 subcomplex at a resolution of 2.60 Å (Supplementary Table S1) and found that this subcomplex assembles into a heterotrimer (Fig. 1b). Consistently, isothermal titration calorimetry (ITC) showed that SMARCC2^(325–518)^ binds to SMARCB1^(169–385)^ following a two-site binding model with two calculated Kd values (Supplementary Fig. S1i).

**Fig. 1:**
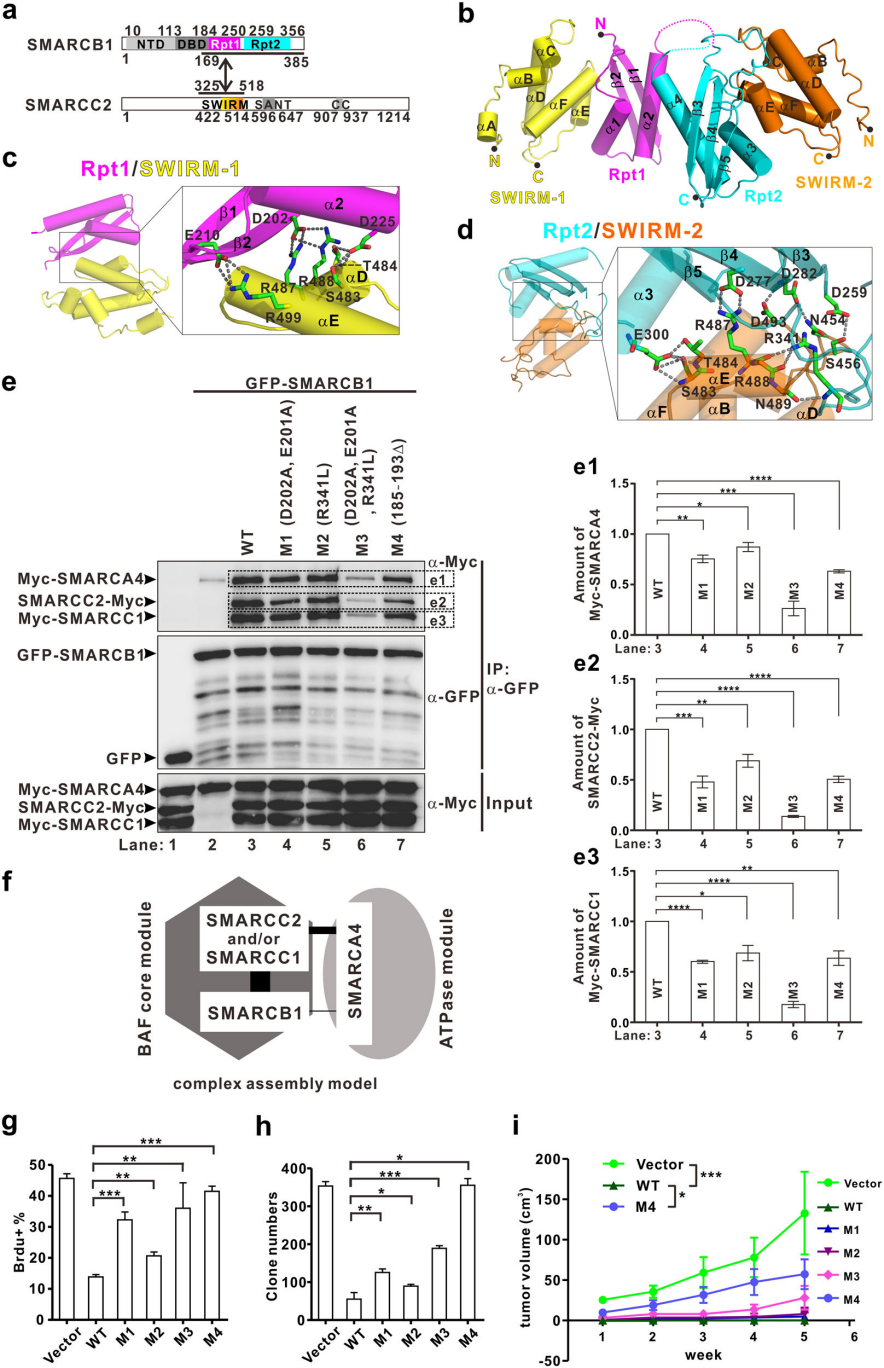
The heterotrimer SMARCB1–SMARCC2 formation is required for the tumor suppression function of SMARCB1. **a** Schematic representations of full-length SMARCB1 and SMARCC2. The protein fragments of the SMARCB1^(169–385)^/SMARCC2^(325–518)^ complex used for structural determination are indicated by a two-way arrow. **b** Ribbon diagram representation of the SMARCB1^(169–385)^/SMARCC2^(325–518)^ complex. **c–d** The interface between Rpt1 or Rpt2 of SMARCB1 and SWIRM of SMARCC2. All interaction details between SMARCB1 and SMARCC2 are shown in Supplementary Fig. S6. **e** Co-IP experiments. The bottom panel shows 3% of the Myc fusion proteins for each IP. **f** Model of SMARCB1–SMARCC1/2–SMARCA4 complex assembly. The filled black hexagon and the filled gray ellipse represent the BAF core and ATPase modules, respectively. For clarify, only SMARCB1, SMARCC1/2, or SMARCA4 are shown in the cartoon diagram. **g**–**h** Statistical graph of the percentage of BrdU positive nuclei (**g**) or the colony number (**h**) in different cell lines. Bars represent average and standard deviation of the percentage of BrdU positive nuclei or the colony number of different cell lines. The experiments were performed in triplicates in **e**–**h**. Error bars represent SEM. **i** Tumor growth curve of nude mice bearing BT-12 cells with inducible expression of SMARCB1 and different mutants in xenograft assay. Data represent mean ± SEM (*n* = 5). *P < 0.05, **P < 0.01, ***P < 0.001, ****P < 0.0001.

In the final model, SMARCB1^(169-385)^ was resolved from aa 184 to aa 356 including the Rpt1 and Rpt2 motifs and SMARCC2^(325–518)^ was well resolved from 423 to 514 including the SWIRM domain (Fig. 1b and Supplementary Fig. S2a-b). However, the N-terminal residues 325–422 of SMARCC2^(325–518)^ lacked observable density, possibly because they were degraded during crystallization (Supplementary Fig. S1j). Rpt1 (consisting of a two-stranded antiparallel β-sheet followed by two α-helices) and Rpt2 (consisting of a three-stranded antiparallel β-sheet and two α-helices) can be superimposed well with a root-mean-square deviation of 0.553 Å for 47 Cα atoms (Fig. 1b and Supplementary Fig. S3a-b). Each Rpt motif binds to a separate SWIRM domain of SMARCC2 to form two separate subcomplexes (Fig. 1b) (defined as Rpt1/SWIRM-1 and Rpt2/SWIRM-2). The SWIRM-1 and SWIRM-2 domains are highly similar to the yeast Swi3-SWIRM domain, respectively (Supplementary Fig. S3c). Overall, the Rpt1/SWIRM-1 complex is very similar to the Rpt2/SWIRM-2 complex and the Rpt1/SMARCC1-SWIRM complex^11^, respectively (Supplementary Fig. S3d). Recently, the cryo-EM sturctures have suggested that the assembly modes for the yeast Snf5-Swi3 and SFH1-RSC8 are similar to those of SMARCB1-SMARCC2 subcomplexes^8–10^. For example, each Rpt motif-mediated complex structure of the SMARCB1^Rpt1-Rpt2^/SMARCC2^SWIRM^ heterotrimeric subcomplex determined in our manuscript is similar to the corresponding portion of the SMARCB1/SMARCC2 subcomplex from the holo-BAF complex (Supplementary Fig. S4).

Interestingly, we noted that the isolated Rpt2 motif, but not the Rpt1 motif, aggregated non-specifically during analytical size-exclusion chromatography (Supplementary Fig. S5a). Consistently, ITC indicated that the Rpt1 motif binds to SMARCC2^SWIRM^ with a Kd of ∼0.12 μM, a value comparable to one of the two Kd values (0.112 μM) measured for SMARCB1^(169–385)^ binding to SMARCC2^(325–518)^ (Supplementary Figs. S1i and S5b). Notably, although the aggregated Rpt2 did not exhibit detectable affinity for SMARCC2^SWIRM^ in ITC (Supplementary Fig. S5c), the Rpt2 motif and SMARCC2^(325–518)^ can form a stable complex when coexpressed (Supplementary Fig. S5d), suggesting that Rpt2 can fold upon binding to SMARCC2^(325–518)^. In addition, an analysis of the circular dichroism spectra showed that the isolated Rpt2 motif possesses no obvious secondary structure (Supplementary Fig. S5e). Thus the Rpt1 and Rpt2 motifs of SMARCB1 bind to the SWIRM domains of two distinct SMARCC2 molecules, promoting the formation a tripartite complex.

Interaction analysis of Rpt1 with SWIRM-1 and Rpt2 with SWIRM-2 reveals multiple sets of hydrogen bonding, hydrophobic, and charge-charge interactions (Fig. 1c, d and Supplementary Fig. S6a-b). Co-immunoprecipitation analysis indicated that the binding of GFP-SMARCB1(D202A, E210A) and GFP-SMARCB1(R341L) to Myc-SMARCC2 is reduced by∼42% and 16%, respectively (Supplementary Fig. S5f). When combined the mutations in Rpt1 and Rpt2 drastically reduced the binding of GFP-SMARCB1(D202A, E210A, R341L) to Myc-SMARCC2 (Supplementary Fig. S5f). Collectively, these results confirm the interaction mode revealed by the structure of the SMARCB1^(169–385)^–SMARCC2^(325–518)^ heterotrimer.

Inspection of the database (http://cancer.sanger.ac.uk/cosmic) identified a total of 64 missense mutations and one in-frame deletion in the segment of SMARCB1 resolved structurally (Supplementary Table S2). Interestingly, an in-frame deletion (185-193Δ) is located at the Rpt1 motif (Supplementary Fig. S7a), suggesting that this in-frame deletion may destroy the folding of Rpt1 motif and therefore the binding of SMARCB1 to SMARCC2. When tested in Co-IP assay, the in-frame deletion only partially impaired the interaction between full-length SMARCB1 and SMARCC2 (Supplementary Fig. S5f). However, when combined with the R341L mutation, the interaction between SMARCB1 and SMARCC2 was completely discrupted (Supplementary Fig. S5g). Consistently, NMR analysis indicated that the deletion (185-193Δ) disrupts the folding of Rpt1 (Supplementary Fig. S5h). Inspection of COSMIC pointed to a total of 25 and 17 missense mutations located in 22 residues of SMARCC2^(423–514)^ and 15 residues of SMARCC1^(447–540)^, respectively (Supplementary Tables S3 and S4). Interestingly, we found that the R487C mutation in SMARCC2 (cognate mutation R512Q in SMARCC1) is located at the interaction interface of the heterotrimer (Supplementary Fig. S7a-b). As anticipated from the structure, biochemical assays indicated that R487C or R512Q reduces the interaction of SMARCC2 or SMARCC1 with SMARCB1, respectively (Supplementary Fig. S7c-f). We found that the cancer-associated R341L mutation of SMARCB1, also located at the interaction interface of the heterotrimer (Supplementary Fig. S7a), affects the interaction between SMARCB1 and SMARCC2 only modestly (Supplementary Fig. S5f). More, we noted that mutations H526P (identified in human congenital hydrocephalus^12^) and R491Q (a cancer-associated) of SMARCC1 map to the SMARCC1^SWIRM^/Rpt1 interface^11^, which potentially disrupt the local folding of the SMARCC1^SWIRM^ domain (Supplementary Fig. S7b and Table S4). The co-IP results showed that the H526P or R491Q mutation disrupts the interaction between SMARCC1 and SMARCB1 (Supplementary Fig. S7g). Two control SMARCC1 mutants (R499C and R499H) efficiently formed a complex with SMARCB1 (Supplementary Fig. S7b and g).

We noted that GFP-SMARCB1 interacts weakly with SMARCA4 in the absence of other subunits and the addition of either SMARCC2 or SMARCC1 substantially increases the association of SMARCA4 with the SMARCB1–SMARCC1/2 subcomplex (Supplementary Fig. S8a). Simultaneous addition of SMARCC2 and SMARCC1 did not further increase the association of SMARCA4 with the SMARCB1–SMARCC1/2 subcomplex (Supplementary Fig. S8a), indicating that SMARCC1 and SMARCC2 may subserve a similar scaffolding function. We next examined the capacity of mutants of SMARCB1, SMARCC1, or SMARCC2 to drive the association of SMARCA4 with the SMARCB1–SMARCC1/2 subcomplex. We found that SMARCB1 mutants M2-(R341L), M1-(D202A, E210A), M4-(Δ185-193), and M3-(D202A, E210A, R341L) exhibit progressively reduced ability to combine with Myc-SMARCA4, SMARCC2-Myc, and Myc-SMARCC1 (Fig. 1e). In addition, there is no influence on the capacity of GFP-SMARCB1 to combine with Myc-SMARCA4 in the context of mutant SMARCC2(R487C) and WT SMARCC1 or mutant SMARCC1(R512Q) and WT SMARCC2 (Supplementary Fig. S8b). However, coexpression of mutants SMARCC2(R487C) and SMARCC1(R512Q) impaired the capacity of GFP-SMARCB1 to associate with Myc-SMARCA4 (Supplementary Fig. S8b). Collectively, these results may partially establish the interaction mode between SMARCB1-SMARCC1/2 and SMARCA4 (Fig. 1f).

To examine the importance of heterotrimer formation for SMARCB1-mediated tumor suppression, we reconstituted the SMARCB1 mutant AT/RT BT-12 cells^13,14^ with doxyciclin-inducible constructs encoding a series of synthetic mutants and one tumor-derived mutation that we had characterized structurally and biochemically. Immunofluorescent staining indicated similar levels of expression and nuclear accumulation of WT and mutants forms of SMARCB1. However, immunioblotting documented somewhat reduced levels of certain SMARCB1 mutants, possibly owing to reduced post-lysis stability (Supplementary Fig. S9a-c). Intriguingly, expression of WT SMARCB1 considerably increased adhesion and spreading, restoring a quasi-normal cell morphology, whereas the tumor-derived mutant M4 and, to a lower extent, M3 did not exert this effect (Supplementary Fig. S9d). All the other mutants exhibited partial phenotypes. Next, BrdU incorporation experiments and plate colony assays indicated that WT SMARCB1 inhibits efficiently the proliferation and colony formation of BT-12 cells, whereas the other four mutants M1–M4 exhibit partial or complete deficient capacity to inhibit cell proliferation and colony formation (Fig. 1g, h and Supplementary Fig. S9e-f). In fact, the inhibitory deficiency of the mutants was proportional to the level of biochemical impairment characterized at the structural and biochemical level.

To examine the mechanisms through which inactivation of SMARCB1 drives malignant rhabdoid tumorigenesis, we conducted RNA-seq studies in BT-12 cells reconstituted with either WT or M4 mutant SMARCB1. GO and Hallmark GSEA indicated that expression of WT but not mutant SMARCB1 downregulates the cell cycle progression genes and upregulates the growth arrest genes, in agreement with the ability of SMARCB1 to suppress proliferation and colony formation. Consistently, these analyses also revealed that SMARCB1 induces expression of several signatures associated with deposition of the extracellular matrix, matrix adhesion, and signaling, as well as the epithelial to mesenchymal transition (Supplementary Figs. S10a-b and S11, and ref. ^15^). The results of transwell assay indicated that WT SMARCB1 promotes both migration and invasion, whereas the mutants exhibit a defect proportional to their level of biochemical impairment (Supplementary Fig. S10c-f). These results indicate that loss of SMARCB1 promotes cell proliferation but also impairs cell adhesion and migration, consistent with the hypothesis that SMARCB1 exerts its tumor suppressive function predominantly by inhibiting proliferation.

We also performed xenotransplantation experiments in nude mice. As anticipated, BT-12 cells transduced with empty vector formed large tumors upon subcutaneous injection in nude mice, whereas those expressing WT SMARCB1 did not (Supplementary Fig. S9g). Interestingly, each of the four mutants M1–M4 exhibited defective capacity to inhibit tumor growth as compared to WT SMARCB1 (Fig. 1i and Supplementary Fig. S9g), indicating that these mutants have lost, at least in part, their tumor-suppressive activity. Collectively, these observations indicate that the formation of the SMARCB1–SMARCC2 subcomplex is required for its subsequent association with SMARCA4 and for tumor suppression.

In conclusion, we determined the crystal structure of the human SMARCB1–SMARCC2 subcomplex and found that this subcomplex assembles into a heterotrimer. The assembly of the subcomplex comprising SMARCB1 and SMARCC2 and/or SMARCC1 is essential for the tumor-suppression function of SMARCB1. Specifically, we propose that the disease-associated mutations in SMARCB1, SMARCC2, and SMARCC1 that impair the formation of the SMARCB1–SMARCC1/2 subcomplex, leading to tumorigenesis.

## Accession codes

Atomic coordinates and structure factors has been deposited in the PDB (6KAG). RNAseq data has been submit NCBI GEO database (GSE139262).

## Supplementary information

Supplementary Information, Figures and Tables

## Supplementary information

**Supplementary Information** accompanies the paper at (10.1038/s41421-020-00196-4).
