# Supplementary material for: A heterotrimeric SMARCB1–SMARCC2 subcomplex is required for the assembly and tumor suppression function of the BAF chromatin-remodeling complex
Source: Cell Discov. 2020 Sep 22;6:66. doi: 10.1038/s41421-020-00196-4 (PMC7506551; doi:10.1038/s41421-020-00196-4)
Supplement: Supplementary file 1 — Supplementary Information, Figures and Tables [file 41421_2020_196_MOESM1_ESM.pdf]

Supplementary information

Materials and methods

Protein expression and purification

To co-express SMARCB1 and SMARCC2, DNA fragments corresponding to human SMARCB1 (full-length residues 1-385) and SMARCC2 (residues 1-958) were polymerase chain reaction (PCR)-amplified from the cDNA library of the Human kidney epithelial (HEK) 293T cell line. SMARCB1 and SMARCC2$^{(1\text{-}958)}$ were cloned into two multiple cloning sites of an in-house modified version of pETDuet-1 vector (Novagen) separately and sequentially. A Thioredoxin (Trx)-his$_6$ tag and a PreScission Protease restriction site are present at the N-terminus of the first and second multiple cloning sites, respectively, in the in-house modified version of pETDuet-1 vector. The resulting proteins contained a Trx-his$_6$ tag on the N-terminus of both SMARCB1 and SMARCC2.

The recombinant proteins were expressed in BL21 (DE3) Codon Plus *Escherichia coli* cells at 16°C for 16–18 h. The cells were then lysed by high pressure cell cracker AH-1500 (ATS Engineering Limited). The Trx-His$_6$-tagged protein complex was purified by Ni-NTA affinity chromatography (QIAGEN) followed by size-exclusion chromatography on a HiLoad 26/60 Superdex 200 (GE Healthcare) in 50mM Tris, pH8.0 and 200mM NaCl. The sodium dodecyl sulphate-polyacrylamide gel electrophoresis (SDS-PAGE) analysis of the recombinant Trx-SMARCB1/Trx-SMARCC2$^{(1\text{-}958)}$ complex is shown in Supplementary Fig. S1c.

The similar co-expression strategies were used to prepare various truncations of the SMARCB1/SMARCC2 complex, residues 169-385 of SMARCB1 and residues 1-958 of SMARCC2 (referred to as Trx-SMARCB1$^{(169\text{-}385)}$/Trx-SMARCC2$^{(1\text{-}958)}$, similar nomenclature hereafter), Trx-SMARCB1$^{(169\text{-}385)}$/Trx-SMARCC2$^{(1\text{-}655)}$, Trx-SMARCB1$^{(169\text{-}385)}$/Trx-SMARCC2$^{(1\text{-}518)}$, SMARCB1$^{(169\text{-}385)}$/SMARCC2$^{(325\text{-}518)}$ and SMARCB1$^{(169\text{-}385)}$/SMARCC2$^{(423\text{-}518)}$. Various mutants were created using standard two-step PCR-based methods and all constructs used in this study were confirmed by DNA sequencing. All of the expression and purification processes were similar to the procedures for the Trx-SMARCB1/Trx-SMARCC2$^{(1\text{-}958)}$ except for SMARCB1$^{(169\text{-}385)}$/SMARCC2$^{(325\text{-}518)}$ and SMARCB1$^{(169\text{-}385)}$/SMARCC2$^{(423\text{-}518)}$ complex proteins, which are purified by several additional steps. After digestion with PreScission Protease to cleave the N-terminal Trx-His_6 tag, the target protein was purified on a Hiprep Q FF 16/10 anion-exchange column. The final purification step was size-exclusion chromatography on a HiLoad 26/60 Superdex 200 column in 50mM Tris, pH8.0 and 200mM NaCl. The SDS-PAGE analyses of all recombinant protein complexes are shown in Supplementary Fig. S1.

The Se-Met-substituted protein was expressed in methionine auxotrophic *E. coli* B834 (DE3) cells grown in LeMaster medium. All of the recombinant proteins were purified by Ni-NTA agarose affinity chromatography followed by ion-exchange and size-exclusion chromatography.

Crystallization and data collection

Both native and selenomethionine (SeMet)-substituted crystals of human SMARCB1$^{(169-385)}$/SMARCC2$^{(325-518)}$ complex were grown at 20 °C at a protein concentration of 15 mg/mL using the sitting drop vapor diffusion method. The protein was equilibrated against a reservoir solution of 150 mM $CsCl_2$, 15% (w/v) PEG 3350 for 30 days. Both crystals were frozen in a cryoprotectant that consisted of the reservoir solution supplemented with 25% (w/v) glycerol. The native crystal diffracted to 2.60 Å with a space group of P4132 and unit cell dimensions of a = b= c = 170.39 Å. The SeMet-substituted crystal diffracted to 2.8 Å in the same space group and with unit cell dimensions of a = b = c = 171.22 Å. All diffraction data were collected at the Shanghai Synchrotron Radiation Facility (SSRF) on beamlines BL17U [1] or BL19U using a CCD detector cooled to 100 K. All datasets were processed and scaled using the HKL2000 software package [2].

Structure determination and refinement

Phasing and initial model building of human SMARCB1$^{(169-385)}$-SMARCC2$^{(325-518)}$ complex crystal structure were determined by single wavelength anomalous dispersion (SAD) using PHENIX AutoSol wizard [3] and AutoBuild wizard [4], respectively. Then, the initial models were further rebuilt and adjusted manually with Coot program [5] and were refined by phenix.refine program of PHENIX [6]. The final model was further validated using MolProbity [7]. Detailed data collection and refinement statistics are summarized in Supplementary Table S1. All structural figures were prepared using PyMOL (http://www.pymol.org/).

Isothermal titration calorimetry

Isothermal titration calorimetry (ITC) measurements were performed on a MicroCal^{TM} Isothermal Titration Calorimeter iTC200 (GE Healthcare) in 50mMTris, pH8.0 and 200mM NaCl. For the protein of SMARCB1$^{(169-385)}$, 88 μM of SMARCC2$^{(325-518)}$ was titrated into 6 μM of SMARCB1$^{(169-385)}$. For the mutant protein of SMARCC2$^{(325-518)}$(R487C), 1.16 mM of SMARCC2$^{(325-518)}$ was titrated into 24.5 μM of SMARCB1$^{(169-385)}$. For the protein of wild-type SMARCC1$^{(353-542)}$, 102 μM of SMARCC1$^{(353-542)}$ was titrated into 12 μM of SMARCB1$^{(169-385)}$. For the mutant protein of SMARCC1$^{(35A3-542)}$(R512Q), 155 μM of SMARCC1$^{(353-542)}$ was titrated into 9.5 μM of SMARCB1$^{(169-385)}$. For the protein of Trx-Rpt1 and Trx-Rpt2, 300 μM of Trx-Rpt1 and 232 μM of Trx- Rpt2 were titrated into 46 μM of Trx-SMARCC2^{SWIRM}, respectively. The titration consisted of an initial injection of 0.4 μl followed by 26 injections of 1.5 μl every 120 s at 25°C. The titration data and binding plot after baseline subtraction were analyzed with the MicroCal Origin software with the two-site and one-site-binding models for SMARCB1$^{(169-385)}$ or SMARCC1$^{(353-542)}$ and SMARCC2$^{(325-518)}$(R487C) or Trx-Rpt1, respectively.

NMR experiments

All NMR spectra of protein samples were recorded at 298 K on a Bruker AV600 NMR spectrometer equipped with a QCI cryoprobe. The protein samples were in 50mM Tris, pH8.0 and 200mM NaCl.

Cell culture and transfection

HEK293T cells were cultured in DMEM (Sigma, USA) supplemented with 10% (vol/vol) FBS (Biological Industries) at 5% CO_2 and 37°C. HEK293T cells were transfected with corresponding plasmids by Polyethylenimine (Polysciences, Inc.) according to the manufacturer's protocol. BT-12 cells were cultured in RPMI 1640 (Gibco, USA) with 10% (vol/vol) FBS (Gibco, USA) at 5% CO_2 and 37°C. To establish SMARCB1 inducible re-expression stable cell lines, BT-12 cells were transduced with lentiviral vector pCW57-GFP-P2A-MCS-Neo-SMARCB1 (addgene, 89181), which can co-express green fluorescent protein (GFP). After 14 day's selection by G418 (Geneticin), cells were treated with doxycycline (1 μg/ml, Sigma-Aldrich) for 3 days to induce the expression of SMARCB1 and various mutants, then GFP positive cells were sorted and maintained in media with doxycycline to perform different assays, including BrdU incorporation assay, plate colony formation, migration and invasion assays, RNA-seq, and xenograft study.

Co-immunoprecipitation

HEK293T cells were transfected with the indicated combinations of plasmids. 24 hours after transfection, HEK293T cells were lysed using ice-cold cell lysis buffer (50 mM Tris-HCl, pH 8, 150 mM NaCl, 8% glycerol, 0.5% NP40, 0.5% Triton X-100, 1 mM phenylmethylsulfonyl fluoride, and protease inhibitor cocktails) and cleared by centrifugation at 13,000 rpm for 20 min at 4°C. The supernatants were then incubated with agarose conjugated anti-GFP antibody for 30 min at 4°C. The agarose beads were washed three times with cell lysis buffer and eluted with SDS sample buffer. Samples were then subjected to SDS-PAGE and western blot analysis.

Western blotting

The proteins were separated by SDS-PAGE and transferred to PVDF membrane (Millipore). The membranes were subsequently blocked with 10% nonfat milk in TBST (50 mM Tris-HCl, pH 7.4, 150 mM NaCl, and 0.1% Tween 20) for 1 h. For the samples of co-IP assays, the membranes were immunoblotted with anti-Myc (Sigma) and anti-GFP (Sigma) antibodies at room temperature for 1 h, and then probed with horseradish-peroxidase-conjugated secondary antibodies (Santa Cruz) and developed with a chemiluminescent substrate (Millipore). Protein bands were visualized on the Tanon-5200 Chemiluminescent Imaging System (Tanon Science and Technology).

BrdU incorporation assay

Anti-BrdU staining was used to measure progression through G1 and entry in S phase. The assays were performed by BrdU Labeling and Detection Kit I (Roche, USA) according to manufacturer's protocol. Briefly, Sorted BT-12 cells were plated at 40,000 cells per well on 8-well chambered slide and were cultured for 24 h. They were then incubated with complete medium supplemented with BrdU for 1 h. After fixation with 100% cold ethanol fixative, cells were stained with anti-Brdu antibody and anti-mouse Alexa Fluor® 555 secondary antibody, then slide were mounted and examined using fluorescence microscope. All experiments were performed in triplicates.

Plate colony formation

Sorted BT-12 cells were dissociated into single cells (1000 /well), were plated into 6-well plates and were allowed to grow for 14 days. The medium was changed every

three days until the cells formed visible clone. Next, the media were removed, cell colonies were fixed by 4% paraformaldehyde and stained with 0.1% crystal violet for 20 min, then cells were washed with water, air dried at room temperature for 1 h, and took a picture by Gel counter (Oxford Optronix, UK). All experiments were performed in triplicates.

Migration and invasion assays

Both migration and invasion assays were performed using a 24-well Transwell chamber system (Corning, USA). Sorted BT-12 cell were plated at 40,000 per well both for cell migration and cell invasion coated with matrigel (300 μg/ml, 1 hr of solidification). Cells were incubated for 24 hr, fixed with 4% paraformaldehyde, stained by 0.1% crystal violet, and took a picture using an inverted microscope. All experiments were performed in triplicates.

RNA-seq and data analysis

Total RNA were extracted from different cell lines using TRIzol (Invitrogen, USA), then 1 μg was send to BGI for quality test and library construction. Libraries were sequenced on a BGISEQ-500. RNA-seq reads were aligned to the human reference genome (hg19) using Tophat (v2.1.1; https://ccb.jhu.edu/software/tophat/index.shtml) [8]. Gene models of Refgene were downloaded from the Illumina's iGenomes project (https://support.illumina.com/sequencing/sequencing_software/igenome.html). FPKM (Fragments Per Kilobase of transcript per Million mapped reads) values were generated using cufflinks (v2.2.1; http://cole-trapnell-lab.github.io/cufflinks/) [9]. Further differential expression analysis was using cuffdiff function which is in cufflinks, and

considered genes with log2 fold change > 4 or < -2 and false discovery rate (FDR) < 0.05 as significantly differentially expressed.

Gene ontology enrichment and Gene set enrichment analysis

Gene ontology (GO) enrichment analysis was done by using Investigate Gene Sets (http://software.broadinstitute.org/gsea/msigdb/index.jsp) [10]. Selected terms from "hallmark gene sets" or "canonical pathways" were considered significant with $p < 0.05$. Gene set enrichment analysis (GSEA) was done by using Genepattern (http://software.broadinstitute.org/cancer/software/genepattern/) [11]. Selected terms from "hallmark gene sets" or "canonical pathways" were considered significant with $p < 0.05$. Next, terms that are both in the top 20 of the GO and the top 20 of the GSEA are selected. Of them, top up/down-regulated genes from the top 4 signatures were selected and their FPKM values between WT SMARCB1 expression cells and M4 mutant expression cells were visualized by Morpheus heatmap tool (https://software.broadinstitute.org/morpheus/).

Xenograft study

All of the procedures related to animal handling, care, and treatment in this study were performed following approval by the Institutional Animal Care and Use Committee of MD Anderson Cancer Center and following the guidelines of the Association for Assessment and Accreditation of Laboratory Animal Care. For the in vivo study, mice were subcutaneously inoculated at the left and right flank with BT-12 tumor cells (1×10^6 cells per injection) in 0.1 mL mixture of media and Matrigel (RPMI

1640/Matrigel, 1:1) for tumor development. Then we used the water containing doxycycline to raise the mice and changed the water containing dox every three days. After five weeks of growth, the mice were sacrificed by euthanasia, and the tumors were removed and photographed.

References

1 Wang, Q. S. *et al.* Upgrade of macromolecular crystallography beamline BL17U1 at SSRF. *Nucl. Sci. Tech.* **29**, 68 (2018).

2 Z, O. & W, M. Processing of X-ray diffraction data collected in oscillation mode. *Methods Enzymol.* **276**, 307-326 (1997).

3 Terwilliger, T. C. *et al.* Decision-making in structure solution using Bayesian estimates of map quality: the PHENIX AutoSol wizard. *Acta Crystallogr. D Biol. Crystallogr.* **65**, 582-601 (2009).

4 Terwilliger, T. C. *et al.* Iterative model building, structure refinement and density modification with the PHENIX AutoBuild wizard. *Acta Crystallogr. D Biol. Crystallogr.* **64**, 61-69 (2008).

5 Emsley, P., Lohkamp, B., Scott, W. G. & Cowtan, K. Features and development of Coot. *Acta Crystallogr. D Biol. Crystallogr.* **66**, 486-501 (2010).

6 Adams, P. D. *et al.* PHENIX: a comprehensive Python-based system for macromolecular structure solution. *Acta Crystallogr. D Biol. Crystallogr.* **66**, 213-221 (2010).

7 Davis, I. W. *et al.* MolProbity: all-atom contacts and structure validation for proteins and nucleic acids. *Nucleic Acids Res.* **35**, W375-383 (2007).

8 Kim, D. *et al.* TopHat2: accurate alignment of transcriptomes in the presence of insertions, deletions and gene fusions. *Genome Biol.* **14**, R36 (2013).

9 Trapnell, C. *et al.* Differential analysis of gene regulation at transcript resolution with RNA-seq. *Nat. Biotechnol.* **31**, 46 (2012).

10 Mootha, V. K. *et al.* PGC-1alpha-responsive genes involved in oxidative phosphorylation are coordinately downregulated in human diabetes. *Nat. Genet.* **34**, 267-273 (2003).

11 Reich, M. *et al.* GenePattern 2.0. *Nat. Genet.* **38**, 500-501 (2006).

Supplementary Figure S1

a

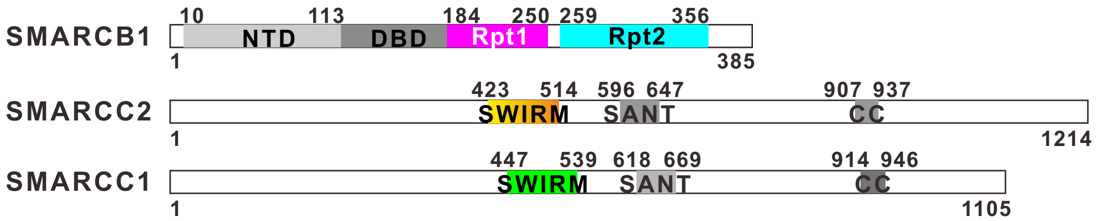

b

Residues of SMARCB1	Residues of SMARCC2	Binding	Supporting data
1-385	1-958	+	Fig. S1C
169-385	1-958	+	Fig. S1D
169-385	1-655	+	Fig. S1E
169-385	1-518	+	Fig. S1F
169-385	423-518	+	Fig. S1G
169-385	325-518	+	Fig. S1H

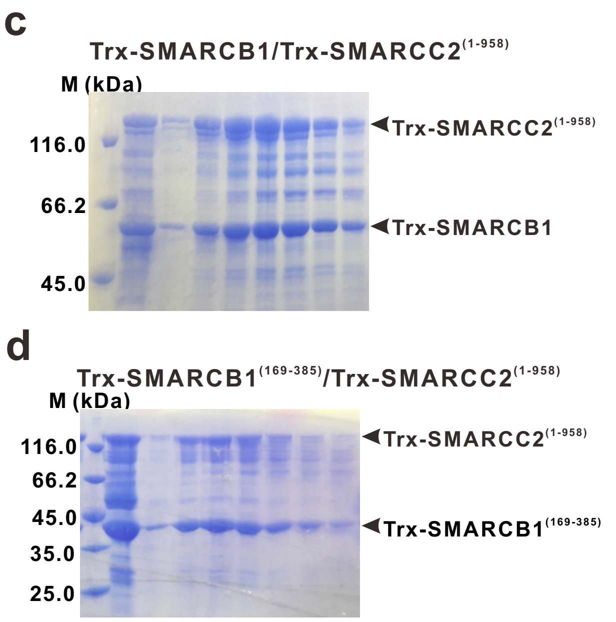

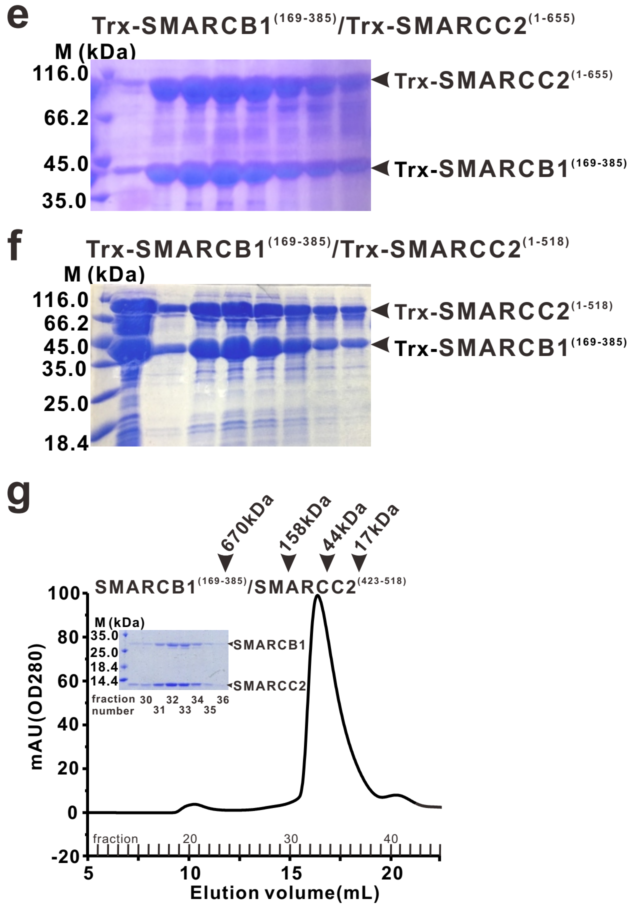

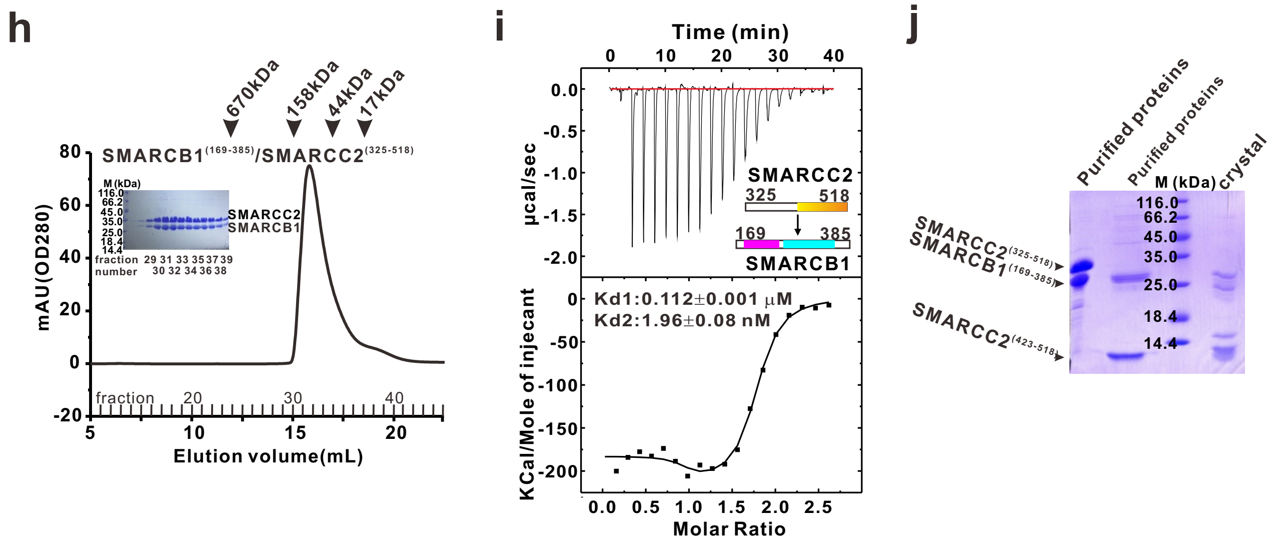

Supplementary Fig. S1. Mapping the minimal reciprocal binding region of the SMARCB1-SMARCC2 subcomplex. (a) Schematic representation of full-length SMARCB1, SMARCC1, and SMARCC2. A N-terminal domain (NTD), a DNA binding domain (DBD), Repeat 1 (Rpt1) (magenta) and Repeat 2 (Rpt2) (cyan) arranged in tandem are shown in SMARCB1. SMARCC1/2 consists of three conserved structural domains: a SWIRM, a SANT, and a CC. **(b)** Gel filtration-based test of the binding of SMARCB1 and SMARCC2 by the co-expression of target proteins. '+' indicates protein binding, according to the supporting data in Supplementary Fig. S1c-h. **(c-f)** SDS-PAGEs of various SMARCB1/SMARCC2 recombinant proteins by co-expression after gel filtration. The analytical gel filtration profiles and the corresponding SDS-PAGEs of SMARCB1$^{(169\text{-}385)}$/SMARCC2$^{(423\text{-}518)}$ and SMARCB1$^{(169\text{-}385)}$/SMARCC2$^{(325\text{-}518)}$ complex are shown in **(g)** and **(h)**, respectively. The elution volumes of the protein markers used to calibrate the gel filtration column are labeled at the top of (**g**) and (**h**). Notably, several recombinant proteins are running at a higher MW in SDS-PAGE than their theoretical MW. (**i**) ITC-based measurement of the binding between SMARCC2$^{(325\text{-}518)}$ and SMARCB1$^{(169\text{-}385)}$. (**j**) SDS-PAGE of the purified SMARCB1$^{(169\text{-}385)}$/SMARCC2$^{(325\text{-}518)}$ or SMARCB1$^{(169\text{-}385)}$/SMARCC2$^{(423\text{-}518)}$ and the SMARCB1$^{(169\text{-}385)}$/SMARCC2$^{(325\text{-}518)}$ proteins dissolved from crystals.

Supplementary Figure S2

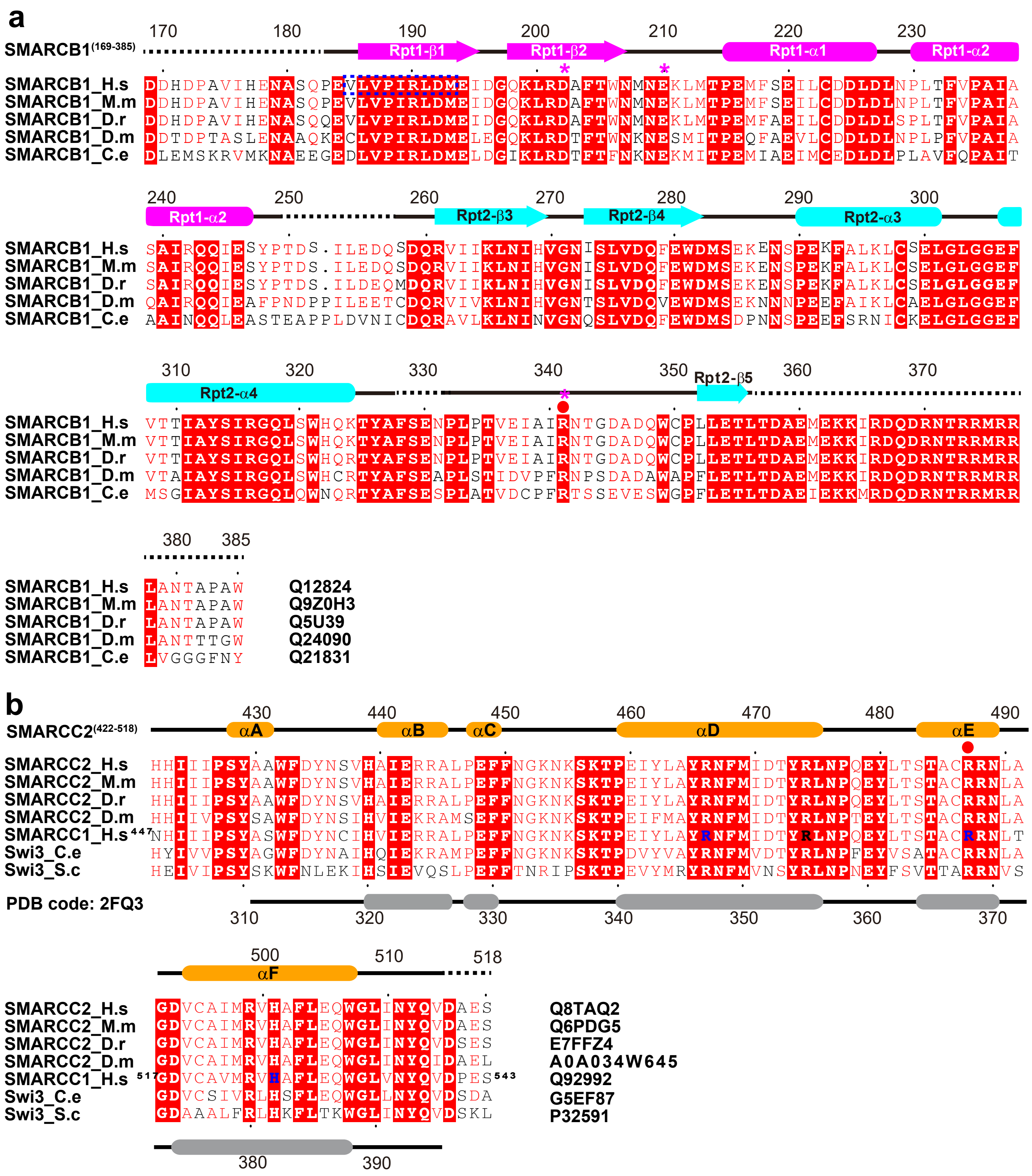

Supplementary Fig. S2. Structure-based sequence alignment of SMARCB1 and SMARCC2 from different species. In this alignment, the secondary structures of SMARCB1 (**a**) or SMARCC2 and Swi3 (**b**) are shown at the top and bottom, respectively, and conserved residues are shaded in red. The residues D202, E210, and R341 of SMARCB1 (**a**), which are absolutely conserved from worm to human and mutated to alanine or leucine, respectively, are indicated with magenta asterisks. The disease-associated amino acids substitutions R341L of SMARCB1 (**a**), R487C of SMARCC2 and R512Q of SMARCC1 (**b**) are indicated with red spheres. The disease-associated amino acids deletion 185-193Δ are indicated with a dotted blue box (**a**). Residues of SMARCC1 involved in disease-associated mutations (R491Q, R499C/H, R512Q, and H526P) are highlighted with blue (**b**). Species abbreviations: H.s, *Homo sapiens*; M.m, *Mus musculus*; D.r, *Danio rerio*; D.m, *Drosophila melanogaster*; C.e, *Caenorhabditis elegans*; S.c, *Saccharomyces cerevisiae*. The GenBank numbers are shown at the end of each alignment.

Supplementary Figure S3

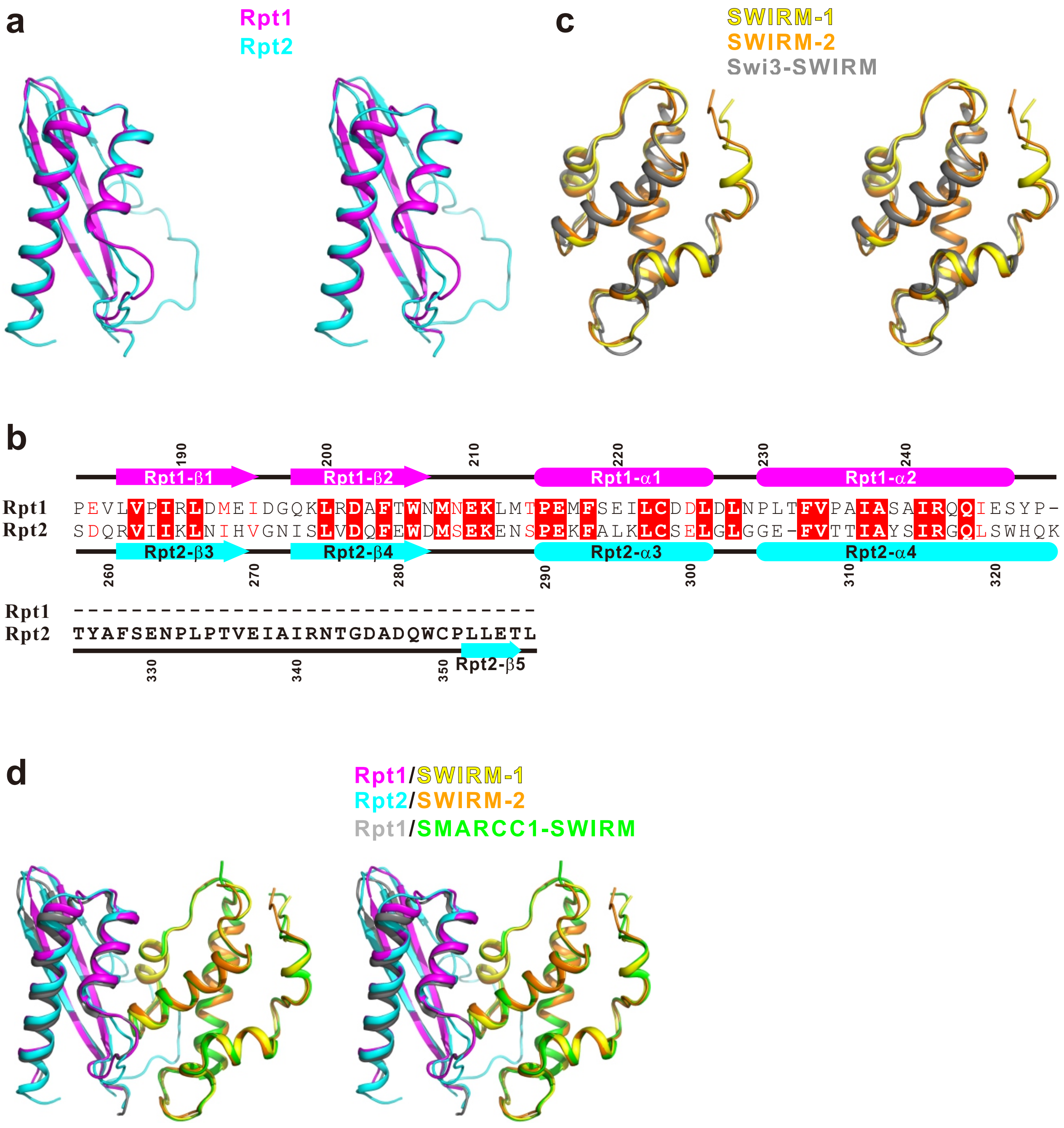

Supplementary Fig. S3. Rpt1 and Rpt2 have similar topology and bind to SWIRM domain with a similar manner. A stereo view of the superimposition of the structure of Rpt1 (magenta) and Rpt2 (cyan) (**a**), SWIRM-1 (yellow) and SWIRM-2 (orange) of SMARCC2 and yeast Swi3-SWIRM (grey) (**c**), or Rpt1/SWIRM-1 complex (magenta and yellow), Rpt2/SWIRM-2 complex (cyan and orange), and Rpt1/SMARCC1-SWIRM complex (grey and green) (**d**). (**b**) Structure-based sequence alignment of Rpt1 and Rpt2. The secondary structures of Rpt1 and Rpt2 are shown at the top and bottom, respectively, and conserved residues are shaded in red.

Supplementary Figure S4

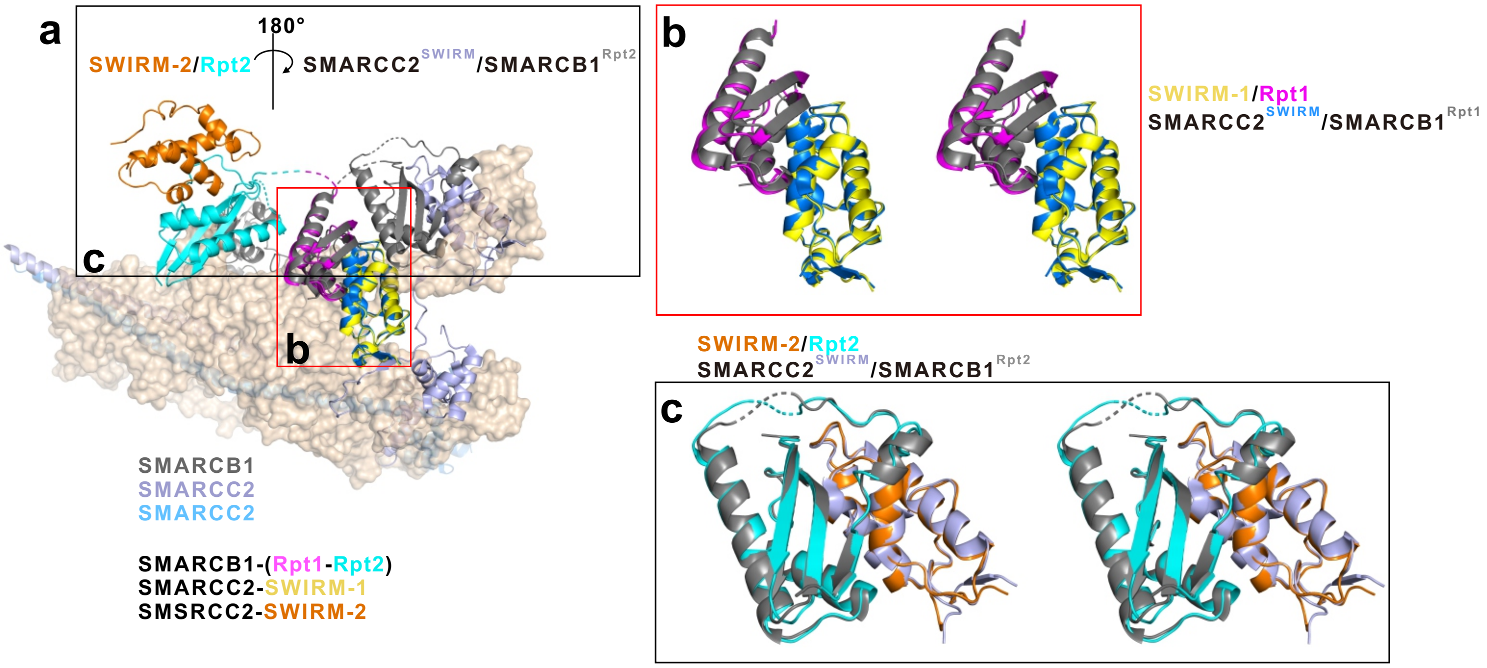

Supplementary Fig. S4. The SMARCB1$^{\text{Rpt1-Rpt2}}$/SMARCC2^{SWIRM} heterotrimer and the corresponding portion of the SMARCB1/SMARCC2 subcomplex from the holo-BAF complex have a similar assembly mode. (**a**) Comparison of the structures of SMARCB1$^{\text{Rpt1-Rpt2}}$/SMARCC2^{SWIRM} heterotrimer (color coded) and SMARCB1/SMARCC2 subcomplex (color coded) in holo-BAF complex (PDB ID 6LTH). For simplify, SMARCB1 and SMARCC2 are shown in cartoon mode and the rest subunits of the holo-BAF complex are shown in surface mode. **(b)** Stereo view of the superimposed structures of the Rpt1 and SWIRM-1 subcomplex determined in this manuscript (magenta and yellow) and SMARCB1$^{\text{Rpt1}}$ and SMARCC2$^{\text{SWIRM-1}}$ subcomplex from the holo-BAF (grey and marine), with a root-mean-square deviation (rmsd) value of 0.652 Å for 151 atoms. **(c)** Stereo view of the superimposed structures of the Rpt2 and SWIRM-2 subcomplex determined in this manuscript (cyan and orange) and SMARCB1$^{\text{Rpt2}}$ and SMARCC2^{SWIRM} subcomplex from the holo-BAF (grey and light blue), with a rsmd value of 0.407 Å for 160 atoms.

Supplementary Figure S5

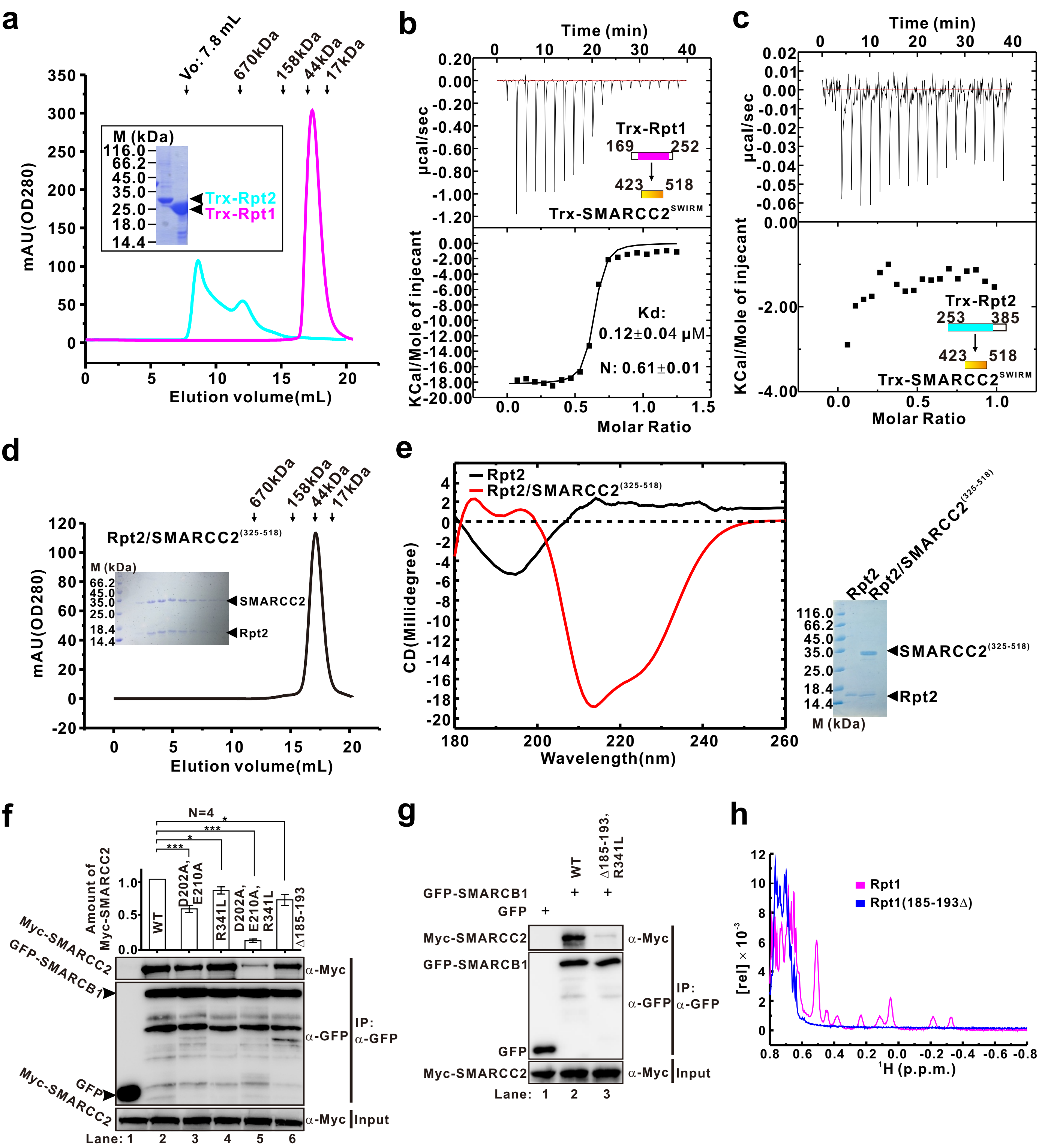

Supplementary Fig. S5. The integral Rpt1-2 tandem in SMARCB1 is essential for binding to the SWIRM domain of SMARCC2 to form a tripartite complex. (**a, d**) Analytical gel filtration profiles and SDS-PAGE of the purified proteins Trx-Rpt1, Trx-Rpt2 and Rpt2/SMARCC2$^{(325\text{-}518)}$ complex. The elution positions for void volume and/or protein markers to calibrate the gel filtration column are indicated at the top. ITC-based measurements of the binding between Trx-SMARCC2^{SWIRM} and Trx-Rpt1 (**b**) or Trx-Rpt2 (**c**). (**e**) The circular dichroism (CD) spectrums of Rpt2 (black curve) and the Rpt2/SMARCC2$^{(325\text{-}518)}$ complex (red curve). First, the purified protein samples were analyzed by SDS-PAGE and then CD spectra of various purified proteins were collected on a MOS450 spectropolarimeter (BioLogic) at room temperature. The protein samples (~ 2.5 μM) were dissolved in 50 mM Tris, pH8.0 and 200 mM NaCl. **(f-g)** Co-IP experiments testing the interaction between SMARCB1 WT or mutants and SMARCC2. Extracts were prepared from HEK293T cells transfected with various combinations of plasmids, as indicated. The bottom panel shows 3% of the Myc fusion proteins for each IP. The error bars indicate the standard error of the mean (SEM) (n = 4, separate experiments). *P < 0.05, ***P < 0.001. (**h**) The deletion (185-193Δ) destroys the folding of Rpt1 by NMR analysis.

Supplementary Figure S6

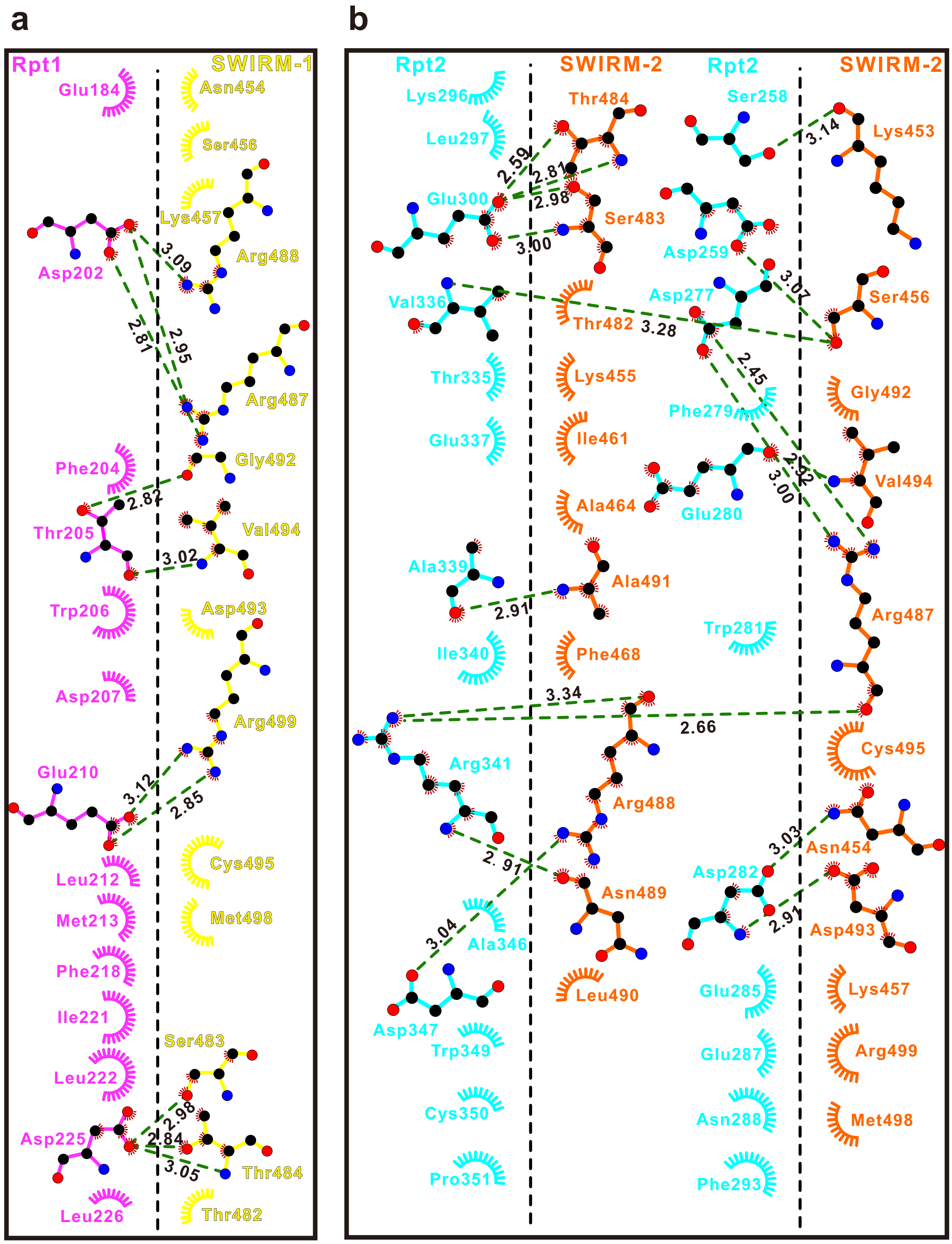

Supplementary Fig. S6. The interaction details between SMARCB1 and SMARCC2. The interaction details between Rpt1 and SWIRM-1 (**a**) and Rpt2 and SWIRM-2 (**b**) are shown. Charge-charge or hydrogen-bonding and hydrophobic interactions are shown as dotted green lines and spoked arcs, respectively.

Supplementary Figure S7

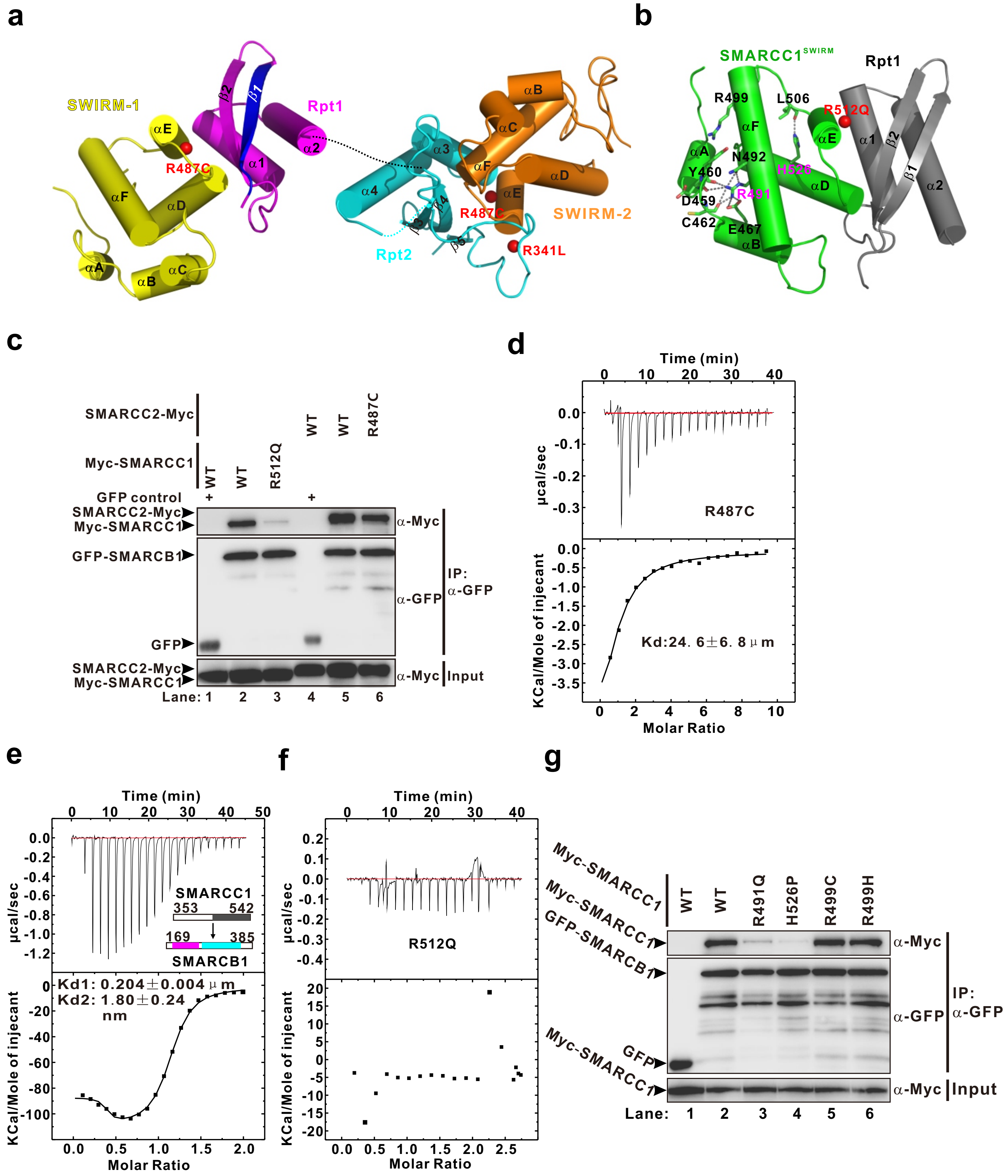

Supplementary Fig. S7. Disease-associated mutations affect the SMARCB1-SMARCC2 or the SMARCB1-SMARCC1 subcomplex formation. (a) Disease-associated mutations in the SMARCB1-SMARCC2 subcomplex. For clarify, only a deletion(185-193Δ) and one missense-mutation site (R341L) of SMARCB1, or one site (R487C) of SMARCC2 are highlighted with blue ribbon and red spheres, respectively. The full lists of disease-associated mutations in SMARCB1 and SMARCC2 are summarized in Supplementary Tables S2 and S3, respectively. **(b)** Ribbon diagram representation of the SMARCB1^{Rpt1}/SMARCC1^{SWIRM} complex structure (PDB ID 5GJK). For clarify, only residues involved in interaction with R491 and H526 (magenta) or R499 are shown, and the side chain are drawn in the stick model. Hydrogen bonding interactions are highlighted by dashed grey lines. A disease-associated mutation R512Q of SMARCC1 is highlighted with red sphere. The full lists of disease-associated mutations in SMARCC1 are summarized in Supplementary Table S4. (**c**, **g**) Co-IP experiments testing the interaction between SMARCB1 and SMARCC2/SMARCC1 WT or mutants. The bottom panel shows 3% of the Myc fusion proteins for each IP. (**d-f**) ITC-based measurements of the binding between SMARCB1 and SMARCC2 or SMARCC1 in the context of their WT or mutations.

Supplementary Figure S8

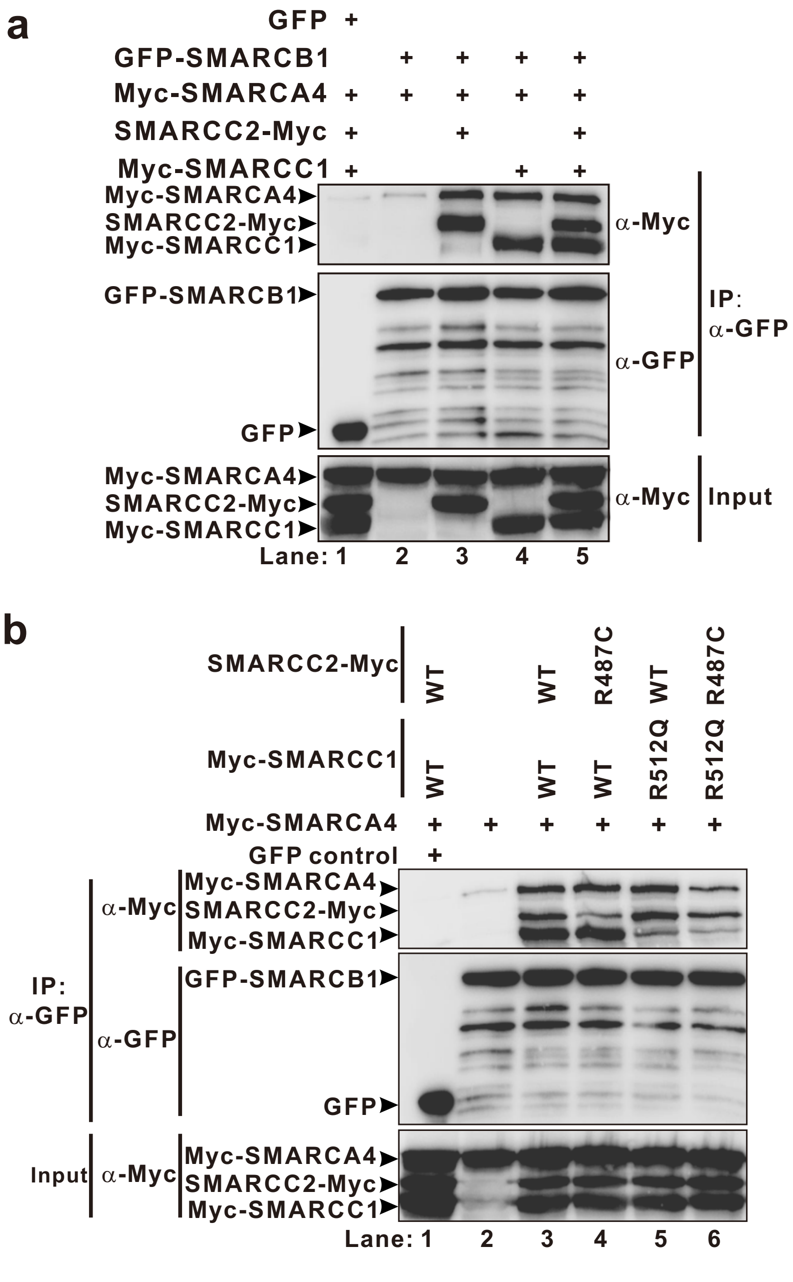

Supplementary Fig. S8. SMARCB1 binds to SMARCA4 through SMARCC2. (a-b) Co-IP experiments. GFP was tagged to the N-terminal of SMARCB1 and Myc was tagged to the N-terminal of SMARCC1 WT or mutant and SMARCA4, or to the C-terminal of SMARCC2 WT or mutant. The bottom panel shows 3% of the Myc fusion proteins for each IP.

Supplementary Figure S9

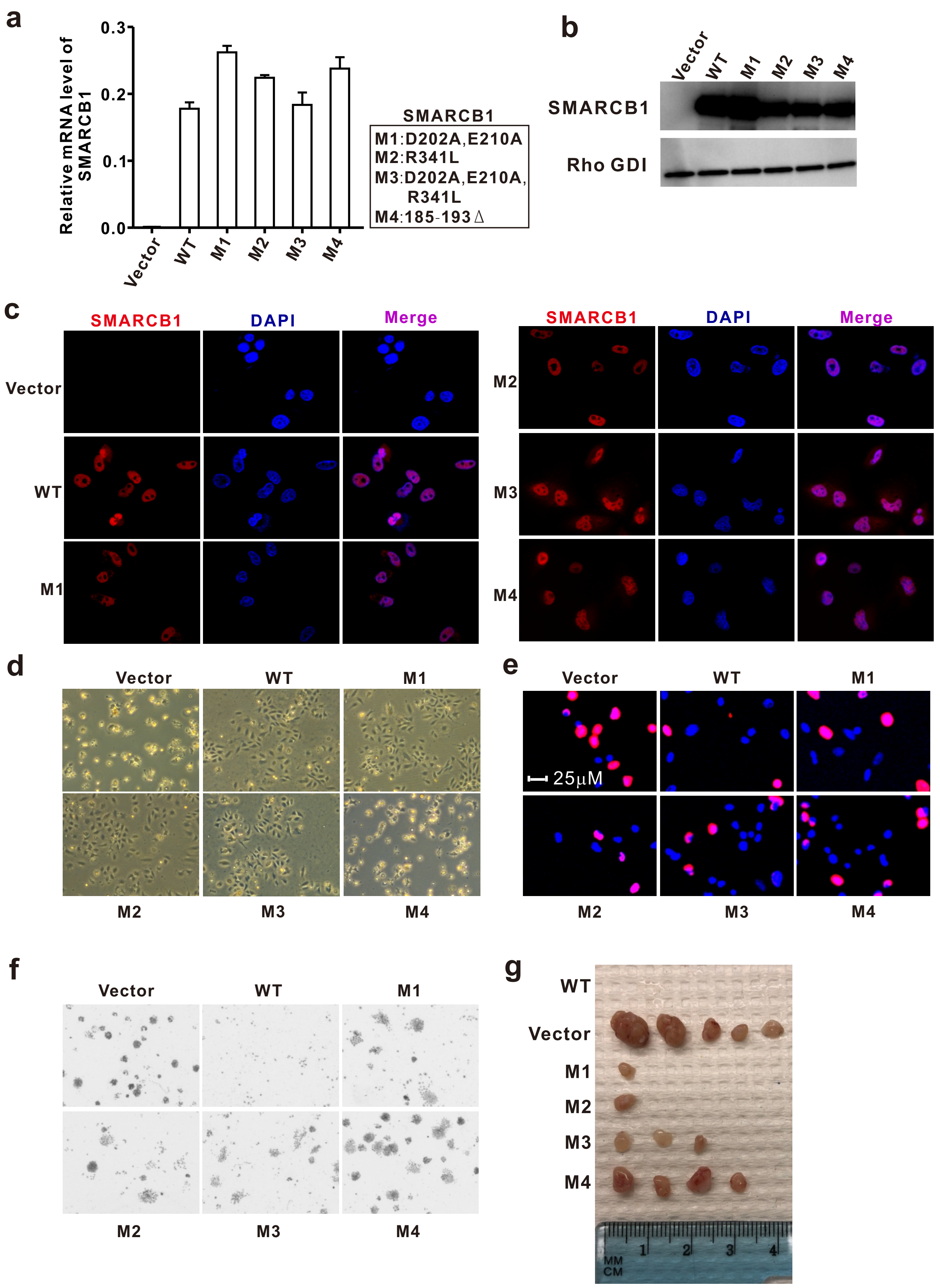

Supplementary Fig.S9. SMARCB1 and SMARCC2 subcomplex is necessary for the tumor-suppression function of SMARCB1. (**a**) The relative mRNA level of SMARCB1 and the different mutants after inducing by doxycycline in BT-12 cells. (**b**) The protein level of SMARCB1 and the different mutants after inducing by doxycycline in BT-12 cells. (**c**) The localization of SMARCB1 in BT-12 cells. SMARCB1 were labeled with Alexa Fluor® 555 (red) and DNA were stained with DAPI (blue). (**d**) The morphology of BT-12 cells were induced expression of SMARCB1 and different mutants. **(e)** BrdU assay of BT-12 cell lines induced expression of SMARCB1 and different mutants. BrdU positive nuclei (red) and DAPI stained nuclei of all the cells (blue) were visualized by fluorescence microscopy. **(f)** Plate colony formation of BT-12 cell lines induced expression of SMARCB1 and the mutants. Colonies were stained with 0.1% crystal violet and took pictures by Gel counter. (**g**) The xenograft assay of BT-12 cell lines with the inducible expression of SMARCB1 and different mutants. Mice were subcutaneously inoculated at the flank with BT-12 cells (1×10^6 cells per injection) in Matrigel and raised by doxycycline for tumor development. After five weeks of growth, the mice were sacrificed and the tumors were removed and photographed.

Supplementary Figure S10

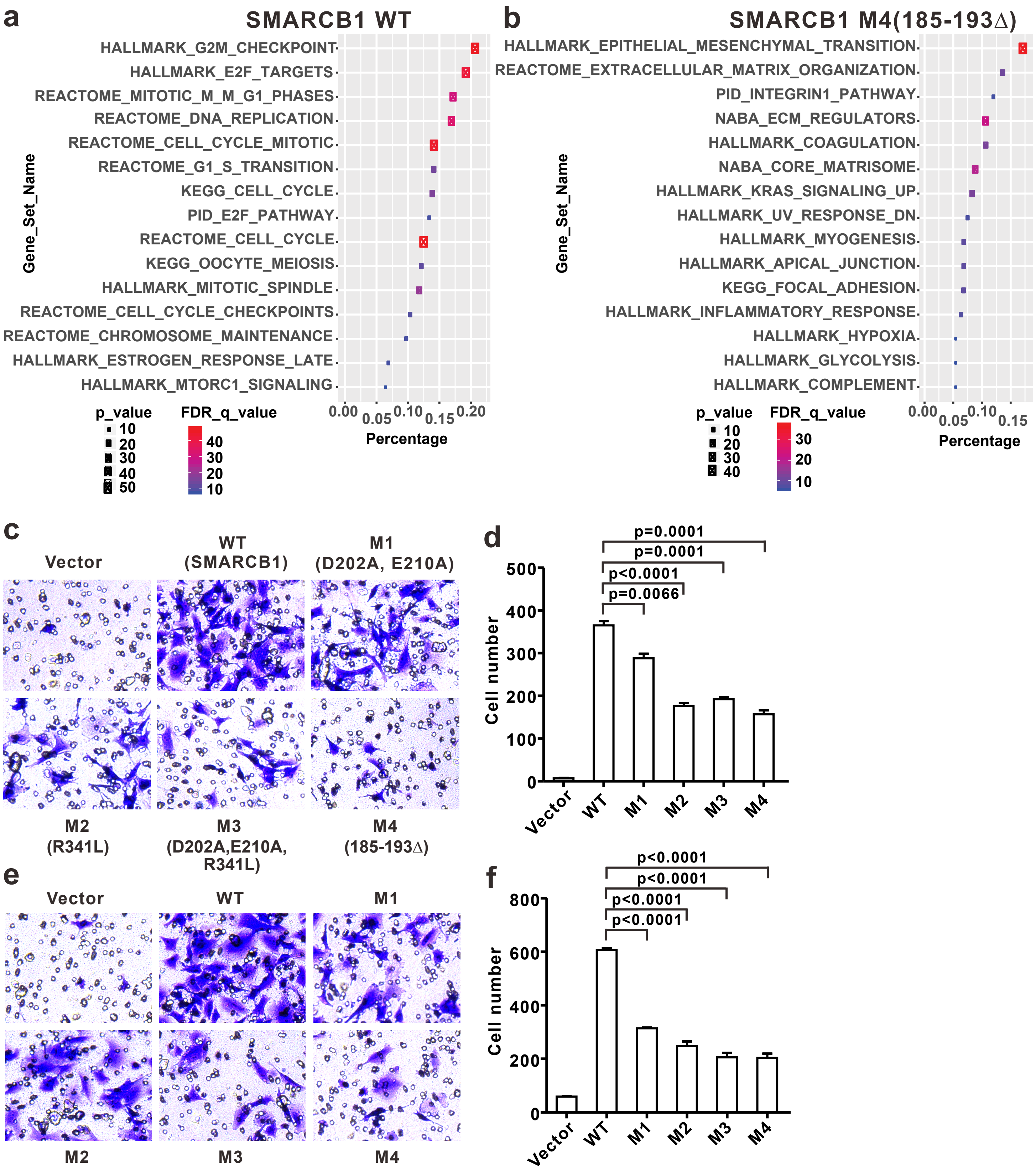

Supplementary Fig.S10. SMARCB1 and SMARCC2 subcomplex is necessary for BT-12 cells migration and invasion. (a-b) Gene set enrichment analysis (GSEA) for BT-12 cells with inducible expression of SMARCB1 (left panel) and M4 mutants (right panel), respectively. Top15 enriched signatures are chosen for each sample. X axis title "Percentage" represents the ratio of number of overlapping genes to the number of genes in signature. Y axis title "Gene_Set_Name" represents the name of each signature. Dots size represents the -log10 p value. Dots color represents the -log10 q_value. (**c, e**) Cell migration and invasion assay of BT-12 cell lines induced expression of SMARCB1 and the mutants, respectively. Cells were stained with 0.1% crystal violet and took pictures by microscopy. **(d, f)** Statistical graph of the cell migration and invasion assay. Bars represent average and standard deviation of the cell number. The experiments were performed in triplicates. Error bars represent SEM (n= 3). *P < 0.05, **P < 0.01, ***P < 0.001.

Supplementary Figure S11

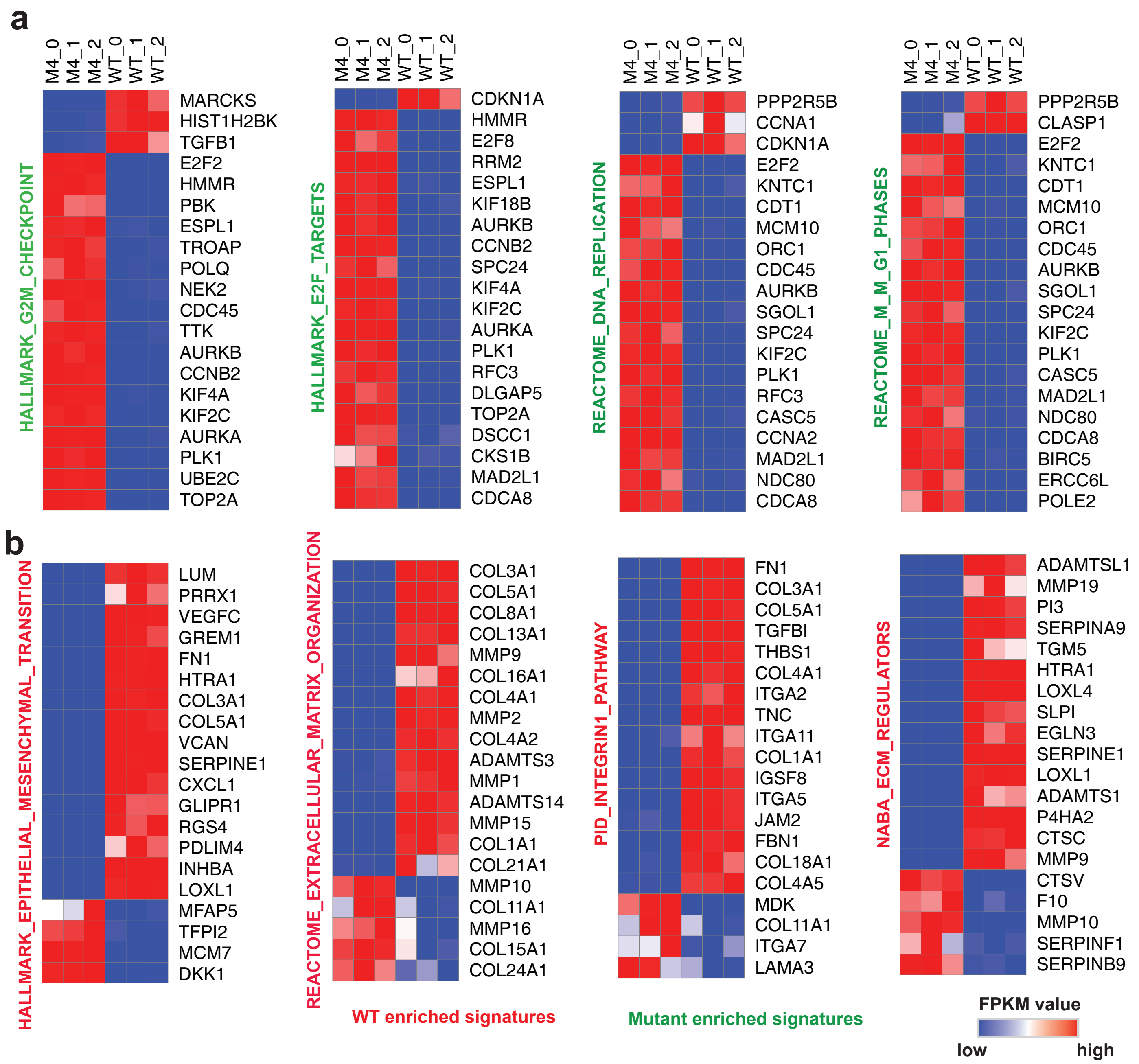

Supplementary Fig. S11. Expression analysis of genes in top4 enriched for M4 mutant cells (a) and SMARCB1 WT cells (b), respectively. Heatmap shows the FPKM value of top up/down genes of each signature. Genes are chosen by their relative expression change (log2 fold change) in M4 mutant cells and SMARCB1 cells.

Supplementary Table S1. Data collection and refinement statistics

**Data collection**	Se-Met-crystal	Wild-type
Space group	***P***4132	***P***4132
Unit cell (Å)	***a***=***b***=***c***=171.22	***a***=***b***=***c***=170.39
Resolution (Å)	50.0-2.80(2.85-2.80) [a]	50.0-2.60(2.69-2.60) [a]
R_{sym} (%)[b]	19.5(110.8) [a]	15.2(94.9) [a]
I/σ	24.0(4.0) [a]	17.1(2.9) [a]
Completeness (%)	100.0(100.0) [a]	100.0(100.0) [a]
Redundancy	46.5(48.1) [a]	12.9(12.4) [a]
**Refinement**		
Resolution (Å)		49.2-2.60
No. of reflections		26,564
$R_{crystal}$ (%)[c]		20.66
R_{free} (%)[d]		24.02
$RMSD_{bond}$ (Å)		0.009
$RMSD_{angle}$(°)		1.002
Number of		
Protein atoms		2,862
Solvent atoms		115
Average B factor ($Å^2$) of		
Protein		51.87

[a] the highest resolution shell.

[b] $R_{sym} = \sum_j \left| \langle I \rangle - I_j \right| / \sum \langle I \rangle$

[c] $R_{crystal} = \sum_{hkl} \left| F_{obs} - F_{calc} \right| / \sum_{hkl} F_{obs}$

[d] R_{free}, calculated the same as $R_{crystal}$, but from a test set containing 5% of data excluded from the refinement calculation

Supplementary Table S2. Disease-associated mutations in SMARCB1

Mutation type	Mutation (Amino acid)
Missence	A5T, K8R, K8N, D22N, D22G, E24Q, E24D, F25L, G29D, E31K, L36P, M38I, M38I, R40Q, G41C, K45E, K45N, Y47S, P48S, R53Q, L54P, T56I, E59D, K62Q, S66L, G69D, T76A, H79Y, G80R, G80E, T82M, A85S, K92N, S94L, E98K, E98Q, D104N, Y107C, K108Q, A109T, A109G, V110G, P116T, P116S, K126N, R127G, P133T, P133L, P136S, H141R, H141L, L142I, P146A, S148F, T149K, N152D, N152S, R155C, R155H, R155L, M156I, G157S, R158Q, R158L, D159N, K160Q, P165L, D169H, D170Y, D172G, P173S, E178K, N179T, A180T, P183L, E184K, V185M, P188S, P188L, R190W, R190Q, R190L, L191M, D192G , R201Q, R201L, D202Y, D202G, D202E, A203T, T205I, N207S, E210K, M213I, P215L, L222I, L222F, P230Q, P230L, T232R, T232M, A238T, A238D, S247C, T250M, S252I, D259N, R261C, R261H, V262I, S274F, F279C, E280K, S284L, E285D, N288K, P290L, A294V, S299L, G305R, F307V, A312T, A312S, S314N, R316W, S320R, T325I, A327T, E330K, E337Q, R341W, R341L, G344S, D347H, P351S, L356P, K363N, R366C, R366P, D367E, Q368R, R370T, R370M, R370S, N371S, T372M, R373G, R373W, R373W, R374W, R374Q, R374P, R374Q, M375I , R376G, R377C, R377H , R377L, R377H , L378F, L378P, A379T, T381A, T381R, A382V, P383L
Nonsense	Q18*, R40*, R40*, Y47*, S49*, S49*, W51*, R53*, Y81*, Q123*, L142*, R158*, Q198*, R201*, W206*, W206*, K211*, E216*, Q243*, Q244*, Q257*, Q260*, K265*, E280*, W281*, W281*, S284*, E306*, Q318*, Y326*, K363*, K364*, Q368*
Frameshift deletion	G33fs*22, L36fs*13, R40fs*15, S42fs*13, Y47fs*22, L50fs*4, R53fs*2, E58fs*12, R60fs*10, I63fs*5, T72fs*13, A93fs*50, E98fs*7, L100fs*5, K108fs*35, I112fs*31, P117fs*26, T118fs*25, T118fs*25, T118fs*25, Y119fs*1, S129fs*36, A144fs*32, L166fs*10, H177fs*32, S181fs*29, Q182fs*27, D196fs*13, K199fs*10, N209fs*4, E210fs*15, C223fs*1, I237fs*30, R242fs*24, Q243fs*37, T250fs*17, D259fs*7, D277fs*79, M283fs*77, P290fs*6, G317fs*3, I340fs*20, G344fs*13, Q348fs*9, P351fs*5, L356fs*4, L356fs*4, R377fs*10, A382fs*5, A382fs*4, P383fs*4, P383fs, P383fs*>3
Frameshift insertion	E24fs*50, K45fs*12, R46fs*25, E59fs*20, S67fs*4, H68fs*3, T72fs*4, L84fs*21, V96fs*10, A109fs*61, S111fs*33, T118fs*52, T118fs*52, T118fs*52, P165fs*6, H171fs*2,

	P183fs*29, L186fs*27, V187fs*26, P188fs*24, I189fs*25, I189fs*24, I189fs*26, L191fs*26, I195fs*20, I195fs*18, D196fs*19, D196fs*29, D196fs*19, A203fs*13, L231fs*56, D251fs*30, D259fs*26, A312fs*9, T357fs*4, M375fs*14, M375fs*12
In-frame deletion	F39delF, T82_A93del12, V185_M193del, L266_*386del, K364delK
In-frame insertion	M4_A5insM

Various disease-associated mutations in SMARCB1 were listed, based on the data extracted from the COSMIC database (http://cancer.sanger.ac.uk/cosmic). A total of 61 missense mutations were located in 48 residues of the well resolved SMARCB1$^{(184\text{-}356)}$ and an in-frame deletion (V185_M193del) was in SMARCB1$^{(184\text{-}356)}$. These 65 mutations were mapped to the modeled structure and divided into four categories: (1) interface of the binding between SMARCB1$^{(169\text{-}385)}$ and SMARCC2$^{(325\text{-}518)}$; (2) folding of SMARCB1$^{(169\text{-}385)}$; and (3) others, and the mutations in each category were colored in red, green, and blue, respectively. Notably, the missense mutations of D259N, R261C, R261H, N288K, R341L, and the in-frame deletion (V185-M193del) affected both the folding of SMARCB1$^{(169\text{-}385)}$ and the interaction between SMARCB1$^{(169\text{-}385)}$ and SMARCC2$^{(325\text{-}518)}$. The mutations used in this study only were shown in Supplementary Fig. S7.

Supplementary Table S3. Disease-associated mutations in SMARCC2

Mutation type	Mutation (Amino acid)
Missence	A18V, T22N, Q23K, R28W, G32R, N34S, E42K, T45I, L49P, Q55H, L56V, P72L, P77L, K85E, S89P, C91F, K101R, R108C, D110N, R119H, M125L, S130F, R139Q, L144M, G154R, G154V, K161N, D170E, N172K, P182S, V191L, R192Q, W215R, H238Y, D244N, T245I, T247I, R268C, K270N, R289W, G294V, S304C, P305S, P305H, S306L, P307L, S321L, T325N, R329C, R329H, P346T, P348S, P350L, N351S, E354Q, P358S, S369L, G374D, D379N, E386K, G392D, E395K, E395Q, N398T, N402S, G404R, K408N, D411N, E414D, H422Q, P427S, A430T, D434E, A440D, A445D, L446V, P447S, N451I, G452S, G452D, A464G, Y465H, N467T, D471H, D471E, Y473C, L475P, P477H, Q478L, A485T, R487C, R487H, L490I, M498T, E505K, A516P, R519Q, M523V, M523K, P526A, P526Q, P526L, T528I, H530D, D536N, V542L, S553F, M558T, F575S, R578H, M581L, V587I, S589F, A597T, R599H, E614K, D619Y, N621H, K622N, G628E, R630H, R630P, R630L, D633N, E634K, H638N, H638D, P643L, E645Q, E653Q, P663S, G670D, A689T, A689V, A692V, F700L, R717Q, A722E, A729V, L735Q, S737R, R750W, E753Q, G755R, G755W, N756K, E768K, E768D, P775L, G778V, E788D, E792D, K795T, E799G, D820N, E822Q, E824G, K828Q, K836N, E840K, G843E, R851W, R851Q, N857S, A861S, A864T, A865T, A870T, S886F, A889T, L890V, L891M, V892M, I901M, K902E, R904W, F906L, R915W, R915L, E918K, R924M, H935Y, A942E, R947W, G968C, P974A, A978T, A978S, V983D, P995S, P1004A, G1008S, P1009L, Q1015K, G1017E, P1037S, G1044R, P1050S, P1058H, M1061T, A1064G, V1073G, A1074T, A1074V, P1078L, G1080D, P1088S, P1091T, A1093P, S1095F, I1106V, A1112V, N1115H, L1116M, F1125Y, G1128S, L1130I, P1133S, A1140V, L1159F, G1160R, P1161S, P1161L, A1167S, A1175T, V1177L, A1201V
Nonsense	Q23*, Q60*, R139*, W190*, R192*, K199*, Q200*, W241*, G294*, E333*, S367*, E419*, Q420*, R466*, R474*, R488*, R519*, Q604*, E614*, Q662*, E811*, Q970*, Q1169*
Frameshift deletion	N11fs*2, E280fs*2, G294fs*46, G780fs*3, P1043fs*18
Frameshift insertion	G65fs*11, L927fs*85, G1044fs*33, G1044fs*18, G1044fs*33
In-frame deletion	N46delN, P974delP, P1092delP, P1092delP, A1112delA

Various disease-associated mutations in SMARCC2 were listed, based on the data extracted from the COSMIC database (http://cancer.sanger.ac.uk/cosmic). A total of 25 missense mutations were located in 22 residues of the well resolved SMARCC2$^{(423\text{-}514)}$. These 25 mutations were mapped into the modeled structure and divided into three categories: (1) interface of the binding between SMARCB1 and SMARCC2; (2) folding of SMARCC2$^{(423\text{-}514)}$; and (3) others, and amino acid substitutions in each category were colored in red, green, and blue, respectively. Only mutations used in this study were shown in Supplementary Fig. S7.

Supplementary Table S4. Disease-associated mutations in SMARCC1

Mutation type	Mutation (Amino acid)
Missence	T12A, V14G, A39V, E44D, A69V, V82L, L84F, H95N, A100T, F109L, W134L, M145L, R147G, R147H, N148I, L180S, L184F, R190Q, Q192E, T196M, A251V, E254K, I258V, D284N, D284E, D288N, R297H, R299G, R323Q, P332S, P334S, T335A, K342N, G347V, R355H, K359E, D370Y, D370V, P374S, E381K, L385I, K392N, D394Y, V400L, V400G, G402V, E410K, E413K, T415A, T417I, G419E, P426S, R433P, S434L, L437R, I450V, S456A, W457C, R469H, Y490H, R491Q, R499C, R499H, R512Q, R513M, L515F, V519G, A521S, A527T, A527V, Q531H, V535I, T553A, P554A, P554S, V558I, V558L, A560V, P563S, L566I, P568L, L569M, H570Y, R572Q, F585I, L601V, R602C, R602H, Y606D, G616V, L632V, K639R, W642C, W642C, N643I, S646W, R652C, T653A, H660Y, L662F, E672K, E672D, S674L, D675N, Q684R, P687F, S691L, N693S, M696V, S697N, D707V, S723F, R724Q, R726W, R726L, E727K, S748P, L773V, E778Q, E782D, D786H, A793V, E800K, E803K, D805E, Q808H, D809Y, D809A, E811D, E826K, E836K, T844A, T844I, D851N, K854N, A869V, A870T, A870P, E889Q, R890T, I892V, K905N, R912Q, I920S, Y949H, A950V, A954T, Q957R, G976A, M991I, P997S, P997L, H1004Y, G1015S, M1023V, G1026A, H1028N, H1028R, R1032L, P1043S, P1043L, S1044F, G1047D, G1052D, L1061V, V1065I, P1092S, P1093L, P1099L, A1101D, S1102L, P1105S
Nonsense	W263*, K345*, S456*, R572*, W623*, E794*, E840*, R953*
Frameshift deletion	R167fs*12, I275fs*3, N281fs*13, N479fs*16, L529fs*1, S564fs*20, D595fs*7, M781fs*44, K814fs*10, N815fs*10
Frameshift insertion	L702fs*34
In-frame deletion	L947_Q960del14

Various disease-associated mutations in SMARCC1 were listed, based on the data extracted from the COSMIC database (http://cancer.sanger.ac.uk/cosmic). A total of 17 missense mutations were located in 15 residues of SMARCC1$^{(447\text{-}540)}$ (PDB ID 5GJK). These 17 mutations were mapped into the modeled structure and divided into three categories: (1) interface the binding between SMARCB1^{Rpt1} and

SMARCC1^{SWIRM}; (2) folding of SMARCC1^{SWIRM}; and (3) others, and amino acid substitutions in each category were colored in red, green, and blue, respectively. Only mutations used in this study were shown in Supplementary Fig. S7.
